# Structure of tri­aqua­tris­(1,1,1-tri­fluoro-4-oxo­pentan-2-olato)cerium(III) as a possible fluorescent compound

**DOI:** 10.1107/S2056989018001135

**Published:** 2018-01-26

**Authors:** Atsuya Koizumi, Takuya Hasegawa, Atsushi Itadani, Kenji Toda, Taoyun Zhu, Mineo Sato

**Affiliations:** a Graduate School of Science and Technology, Niigata University, 8050 Ikarashi 2-nocho, Niigata 950-2181, Japan; b Department of Marine Resource Science, Faculity of Agriculture and Marine Science, Kochi University, 200 Otsu, Monobe, Nankoku City, Kochi 783-8502, Japan; c Center for Advanced Marine Core Research, Kochi University, Nankoku 783-8502, Japan; d Department of Human Sciences, Obihiro University of Agriculture and Veterinary Medicine, Inada-cho, Obihiro, Hokkaido 080-8555, Japan; e Nenjiang Senior High School, Nenjiang Heihe City, Heilongjiang Province, 161400, People’s Republic of China; f Department of Chemistry and Chemical Engineering, Faculty of Engineering, Niigata University, Ikarashi 2-no-cho, Niigata City, 950-2181, Japan

**Keywords:** crystal structure, 1,1,1-tri­fluoro­acetyl­acetone, cerium complex, fluorescence

## Abstract

Luminescence due to the *d–f* transition of Ce^3+^ is quite rare in metal–organic complexes where concentrate quenching frequently occurs. One of the possible ways to avoid this is to design an architecture with elongated metal–metal distances. In the structure of the title complex, tri­aqua­tris­(1,1,1-tri­fluoro-4-oxo­pentan-2-olato-κ^2^ *O*,*O*′)cerium(III), [Ce(C_5_H_4_F_3_O_2_)_3_(H_2_O)_3_], the Ce^III^ complex is linked to neighbouring ones by hydrogen bonding. Within the complex, the Ce^III^ atom is coordinated by nine O atoms from three 1,1,1-tri­fluoro-4-oxo­pentan-2-olate (tfa) anions as bidentate ligands and three water mol­ecules as monodentate ligands. Thus, the coordination number of Ce^III^ atom is nine in a monocapped square–anti­prismatic polyhedron. The F atoms of all three independent CF_3_ groups in tfa are disordered over two positions with occupancy ratios of about 0.8:0.2. The inter­molecular hydrogen bonds between the ligands involve tfa–water inter­actions along the [110] and [1-10] directions, generating an overall two-dimensional layered network structure. The presence of the F atoms in the tfa anion is responsible for an increased inter­molecular metal–metal distance compared to that in the analogous acetyl­acetonate (acac) derivatives. Fluorescence from Ce^3+^ is, however, not observed.

## Abstract 2

The title complex has two kinds of ligands, tri­fluoro­acetyl­acetonate and water. As a result of the presence of F atoms in the 1,1,1-tri­fluoro-4-oxo­pentan-2-olate ligand, the metal–metal distance is longer than in the case of the analogous yttrium complex containing an acetyl­acetonato ligand and also for the analogous lanthanum complex containing acetyl­acetonate.

## Chemical context   

*β*-diketonate ligands have been used widely in metal–organic complexes involving rare earth elements because of their simple usage as organic bidentate ligands (Binnemans, 2005 ▸). The nature of the ligand used is important for a possible enhancement of the luminescence efficiency and intensity; for example, acac is known to have a possible effect on the 4*f*–4*f* transition emission of Eu^3+^ (Kuz’mina *et al.*, 2006 ▸). Tb(acac)_3_ was first used as an active light-emitting layer material in LEDs based on the emission from the lanthanide complex (Kido *et al.*, 1990 ▸). Recently, a lanthanide complex containing Tb^3+^ and Eu^3+^, hexa­fluoro­acetyl­acetonate (hfa) and 4,4′-bis­(di­phenyl­phosphor­yl)biphenyl (dpdp), [Tb_0.99_Eu_0.01_(hfa)_3_(dpdp)]_*n*_, was reported to exhibit an expression thermo-sensing emission, called *chameleon* luminophore (Miyata *et al.*, 2013 ▸; Hasegawa & Nakanishi, 2015 ▸). The hfa anion can absorb efficiently a visible light excitation and transfer the excited energy from hfa to Tb^3+^, because the energy of the triplet state of hfa (22 000 cm^−1^) is very close to an energy level of Tb^3+^ (20 500 cm^−1^; Katagiri *et al.*, 2004 ▸). However, the proximity of the levels causes a back-energy transfer from Tb^3+^ to hfa. The probability of three types of energy transfer from hfa to Tb^3+^, from Tb^3+^ to Eu^3+^ and from Tb^3+^ to hfa is temperature dependent. As a result, the complex can show green, yellow, orange and red emissions despite the 4*f*–4*f* transition.
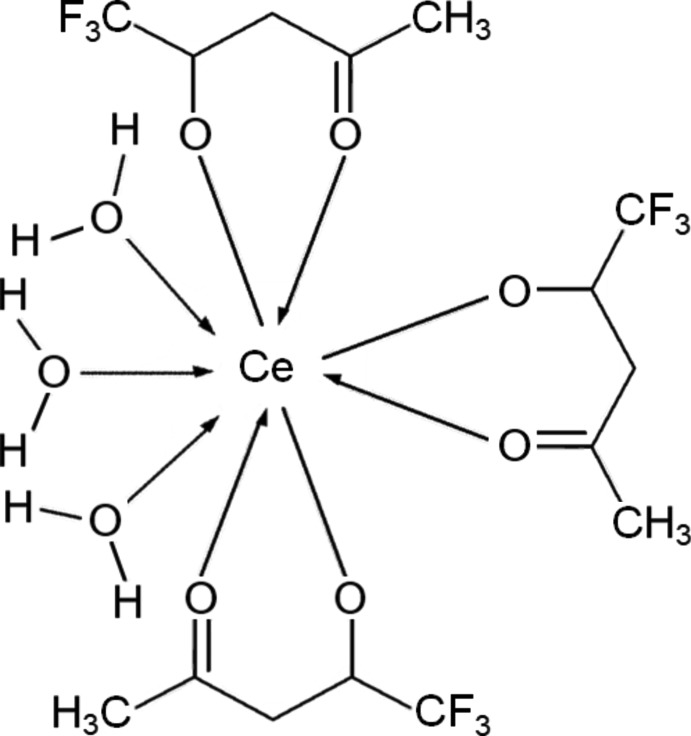


The nature of the ligand is important in the design of fluorescent metal–organic complexes. The F atoms in hfa are larger than the H atoms in acac, which means that the hfa ligand can reduce the energy loss due to thermal vibrations and could increase the inter­molecular distance between the central lanthanide atoms. This may control the concentration quenching.

A considerable number of metal–organic complexes containing Ce^3+^ have been reported so far, but the examples of emission based on the 5*d*–4*f* transition of Ce^3+^ in metal–organic complexes are scarce. [Ce(triRNTB)_2_](CF_3_SO_3_)_3_ [NTB = *N*-substituted tris­(*N*-alkyl­benzimidazol-2-ylmeth­yl)amine] and _∞_^3^[Ce(Im)_3_(ImH)]·ImH (Zheng *et al.*, 2007 ▸; Meyer *et al.*, 2015 ▸) are some of the rare cases. One of the reasons for the small number of fluorescent metal–organic complexes containing Ce^3+^ is the too short distance between the Ce^3+^ ions, causing luminescence quenching by the energy transfer between Ce^3+^ ions. [Ce(triRNTB)_2_](CF_3_SO_3_)_3_ can show a blue emission thanks to a long Ce—Ce distance of about 17–18 Å. The use of more bulky ligands such as NTB is favourable for a longer Ce—Ce distance. _∞_^3^[Ce(Im)_3_(ImH)]·ImH also shows a blue fluorescence emission despite a relatively short separation between the Ce^3+^ cations of 7 Å. Emission occurs more frequently in 3D structures with isolated complexes than in framework structures.

This study reports structural data on a newly synthesized Ce^3+^ complex with functional ligands of tfa.

## Structural commentary   

**Figure 1:**
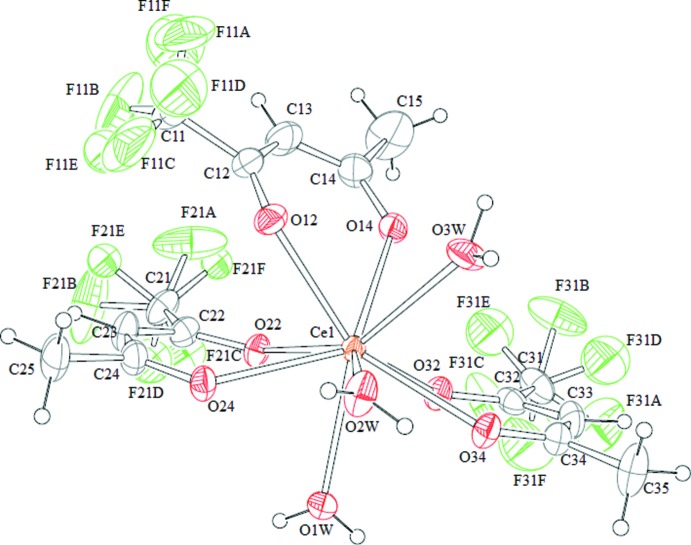
View of the mol­ecular structure of the title complex, with displacement ellipsoids for non-H atoms drawn at the 30% probability level.

The title complex crystallizes in the ortho­rhom­bic space group *Pcab* with eight formula units of [Ce(C_5_F_3_H_4_O_2_)_3_(H_2_O)_3_]. Each mol­ecule is isolated individually, *i.e.* the crystal structure is not a framework structure. The central Ce atom is coord­in­ated by nine O atoms of three hfa and three water mol­ecules (Fig. 1 ▸). Thus, the Ce atom has a monocapped square–anti­prismatic coordination. The Ce—O bond lengths can be classified into two categories; the first is involved in inter­actions with a bidentate hfa, and the second in inter­actions with monodentate water mol­ecules. All distances are comparable with those reported for tfa complexes (Nakamura *et al.*, 1986 ▸). The tri­fluoro­methyl groups of tfa coordinating the Ce^3+^ ion are all disordered on the F atoms, as is frequently observed in tri­fluoro­acetate and tetra­fluoro­borate complexes (Hamaguchi *et al.*, 2011 ▸; Strehler *et al.*, 2015 ▸).

## Supra­molecular features   

**Figure 2:**
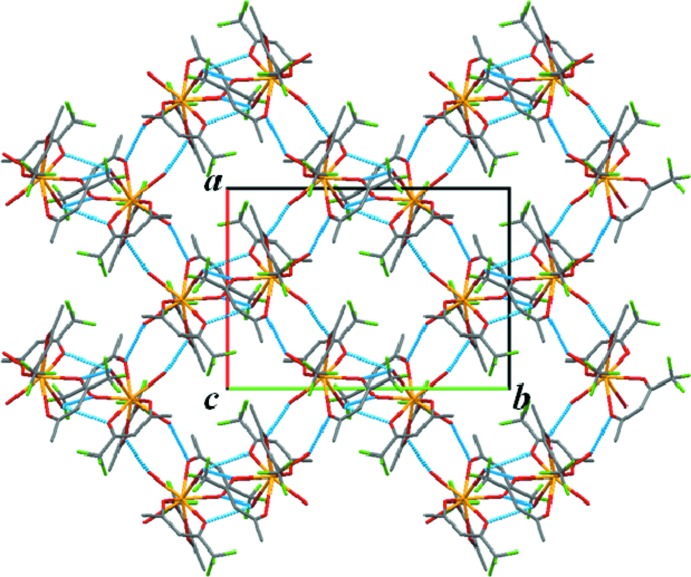
Connection of discrete complexes by inter­molecular hydrogen-bonded (blue lines) chains in the *ab* plane, viewed in projection along the *c* axis. Colour code: Ce yellow, C grey, F green and O red. H atoms have been omitted. Only the major components of the disordered CF_3_ groups are shown for clarity.

**Figure 3:**
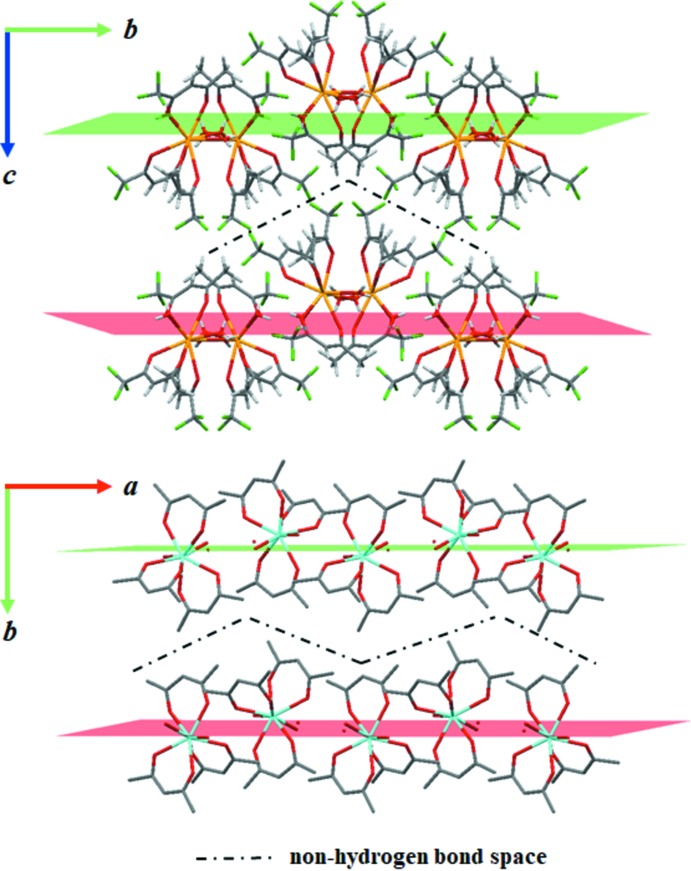
Comparison of the layered structures of the title compound and that of the [Y(C_*5*_H_7_O_2_)_3_(H_2_O)_3_] complex (Cunningham *et al.*, 1967 ▸). Colour code: Ce yellow, Y light blue, C grey, F green and O red. H atoms have been omitted. Only the main components of the disordered CF_3_ groups are shown for clarity.

The individual complexes are linked to neighbouring ones by four types of hydrogen bonds (Table 1 ▸), nearly within the *ab* plane. There are two types of hydrogen-bond directions; the first are parallel to [110] and the second are parallel to [1
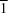
0]. The chains consisting of the complex mol­ecules and the hydrogen bonds, two types of which are cross-linked to each other, building up two-dimensional networks (Fig. 2 ▸). The functional hydro­phobic groups of –CF_3_ and –CH_3_ are located on the outside of the layer, resulting in the stabilization of the stacking layers by inter­molecular forces. Such a layer structure is also observed in the acetyl­acetonate complex, [Y(C_*5*_H_7_O_2_)_3_(H_2_O)_3_] (Cunningham *et al.*, 1967 ▸) (Fig. 3 ▸). This yttrium complex also contains an isolated water in the structure, different from the title compound, but the water mol­ecule can act as a hydrogen-bond linker because it exists within a mol­ecular layer. As a result, the hydrogen bonds make a two-dimensional layered network, as in the title compound. The *Ln*—*Ln* distance of nearest neighbours in this complex is longer than that of [Y(C_*5*_H_7_O_2_)_3_(H_2_O)_3_], the shortest distance in the former being 6.141 Å and in the latter 6.035 Å. This difference is mainly caused by atomic size difference between F and H atoms, even taking into account the atomic size difference between La and Y. The shortest *Ln*—*Ln* distance of [La(C_5_H_7_O_2_)_2_(C_3_H_4_N_2_)(NO_3_)(H_2_O)_2_] (6.247 Å; Koizumi *et al.*, 2017 ▸) is slightly longer than that of the title compound. The fact that the present complex does not show any luminescence from Ce^3+^ can certainly be attributed to an insufficient metal–metal separation. Based on previous studies and the present work, the minimum metal–metal separation is expected to be more than 7 Å.

## Database survey   

Crystal structures of related complexes involving lanthanide ions have been reported with acac ligands (Berg & Acosta, 1968 ▸; Binnemans, 2005 ▸; Filotti *et al.*, 1996 ▸; Fujinaga *et al.*, 1981 ▸; Lim *et al.*, 1996 ▸; Phillips *et al.*, 1968 ▸; Richardson *et al.*, 1968 ▸; Stites *et al.*, 1948 ▸), with tfa complexes (Ilmi *et al.*, 2015 ▸; Katagiri *et al.*, 2007 ▸; Li *et al.*, 2017 ▸; Lim *et al.*, 1996 ▸; Nakamura *et al.*, 1986 ▸; Yan *et al.*, 2009 ▸) and with hfa complexes (Subhan *et al.*, 2014 ▸; Fratini *et al.*, 2008 ▸; Hasegawa *et al.*, 2013 ▸, 2015 ▸; Kataoka *et al.*, 2016 ▸; Rybkin *et al.*, 2011 ▸; Tsaryuk *et al.*, 2017 ▸; Wang *et al.*, 2017 ▸; Yuasa *et al.*, 2011 ▸).

## Synthesis and crystallization   

Yellow plate-like crystals were obtained by slow evaporation from an acetone solution of Ce(NO_3_)_3_·6H_2_O and tri­fluoro­acetyl­acetone (1:3 molar ratio). The products were filtered off and dried at room temperature.

## Refinement   

Crystal data, data collection and structure refinement details are summarized in Table 2 ▸. H atoms bonded to a C atom were positioned geometrically after each cycle in idealized locations and refined as riding on their parent C atoms with C—H = 0.93 Å and *U*_iso_(H) = 1.2*U*_iso_(C atom). All hydrogen atoms bonded to a water O atom were located in a difference-Fourier map and refined isotropically with a distance restraint of 0.85 (2) Å and with thermal restraints *U*_iso_(H) = 1.5*U*_iso_(O atom). The occupancies of the disordered F atoms in the –CF_3_ group were refined for the pairs F11*A*/F11*D*, F11*B*/F11*E* and F11*C*/F11*F* to be 0.829 (14)/0.171 (14), for the pairs of F21*A*/F21*F*, F21*B*/F21*E* and F21*C*/F21*F* to be 0.838 (17)/0.162 (17), and for the pairs of F31*A*/F31*D*, F31*B*/F31*E* and F31*C*/F31*F* to be 0.836 (11)/0.164 (11).

## Supplementary Material

Crystal structure: contains datablock(s) global, I. DOI: 10.1107/S2056989018001135/vn2133sup1.cif

Structure factors: contains datablock(s) I. DOI: 10.1107/S2056989018001135/vn2133Isup2.hkl

CCDC reference: 1817747

Additional supporting information:  crystallographic information; 3D view; checkCIF report

## Figures and Tables

**Table 1:** Hydrogen-bond geometry (Å, °)

*D*—H⋯*A*	*D*—H	H⋯*A*	*D*⋯*A*	*D*—H⋯*A*
O1*W*—H1*WA*⋯O32^i^	0.84 (2)	2.13 (3)	2.927 (4)	158 (5)
O1*W*—H1*WB*⋯O22^i^	0.83 (2)	2.23 (4)	2.969 (4)	149 (6)
O2*W*—H2*WA*⋯O14^ii^	0.85 (2)	1.91 (2)	2.759 (4)	177 (6)
O3*W*—H3*WA*⋯O24^iii^	0.85 (2)	1.94 (2)	2.792 (4)	176 (7)

Symmetry codes: (i) 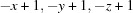; (ii) 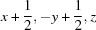; (iii) 
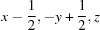
.

**Table 2:** Experimental details

Crystal data	
Chemical formula	[Ce(C_5_H_4_F_3_O_2_)_3_(H_2_O)_3_]
*M*_r_	653.41
Crystal system, space group	Orthorhombic, *P* *c* *a* *b*
Temperature (K)	293
*a*, *b*, *c* (Å)	11.6347 (7), 16.5121 (9), 24.5577 (17)
*V* (Å^3^)	4717.9 (5)
*Z*	8
Radiation type	Mo *K*α
μ (mm^−1^)	2.04
Crystal size (mm)	0.3 × 0.19 × 0.11

Data collection	
Diffractometer	Rigaku XtaLAB mini
Absorption correction	Multi-scan (*REQAB*; Rigaku, 1998 ▸)
*T*_min_, *T*_max_	0.603, 0.805
No. of measured, independent and observed [*I* > 2σ(*I*)] reflections	45156, 5404, 4309
*R*_int_	0.039
(sin θ/λ)_max_ (Å^−1^)	0.649

Refinement	
*R*[*F* ^2^ > 2σ(*F* ^2^)], *wR*(*F* ^2^), *S*	0.036, 0.092, 1.11
No. of reflections	5404
No. of parameters	367
No. of restraints	60
H-atom treatment	H atoms treated by a mixture of independent and constrained refinement
Δρ_max_, Δρ_min_ (e Å^−3^)	0.78, −0.44

Computer programs: *CrystalClear* (Rigaku, 2006 ▸) and *SORTAV* (Blessing, 1995 ▸), *SIR2014* (Burla *et al.*, 2015 ▸), *SHELXL2014* (Sheldrick, 2015 ▸) and *ORTEP-3 for Windows* and *WinGX* (Farrugia, 2012 ▸).

## supplementary crystallographic information

### Crystal data

**Table:**

[Ce(C_5_H_4_F_3_O_2_)_3_(H_2_O)_3_]	*F*(000) = 2552
*M**_r_* = 653.41	*D*_x_ = 1.84 Mg m^−^^3^
Orthorhombic, *P**c**a**b*	Mo *K*α radiation, λ = 0.71073 Å
Hall symbol: -P 2bc 2ac	Cell parameters from 36866 reflections
*a* = 11.6347 (7) Å	θ = 3.0–27.5°
*b* = 16.5121 (9) Å	µ = 2.04 mm^−^^1^
*c* = 24.5577 (17) Å	*T* = 293 K
*V* = 4717.9 (5) Å^3^	Prism, yellow
*Z* = 8	0.3 × 0.19 × 0.11 mm

### Data collection

**Table:**

Rigaku XtaLAB mini diffractometer	5404 independent reflections
Radiation source: sealed x-ray tube	4309 reflections with *I* > 2σ(*I*)
Graphite monochromator	*R*_int_ = 0.039
Detector resolution: 10 pixels mm^-1^	θ_max_ = 27.5°, θ_min_ = 3.0°
phi or ω oscillation scans	*h* = −14→15
Absorption correction: multi-scan (*REQAB*; Rigaku, 1998)	*k* = −21→21
*T*_min_ = 0.603, *T*_max_ = 0.805	*l* = −31→31
45156 measured reflections	

### Refinement

**Table:**

Refinement on *F*^2^	Primary atom site location: structure-invariant direct methods
Least-squares matrix: full	Secondary atom site location: difference Fourier map
*R*[*F*^2^ > 2σ(*F*^2^)] = 0.036	Hydrogen site location: mixed
*wR*(*F*^2^) = 0.092	H atoms treated by a mixture of independent and constrained refinement
*S* = 1.11	*w* = 1/[σ^2^(*F*_o_^2^) + (0.0377*P*)^2^ + 9.6633*P*] where *P* = (*F*_o_^2^ + 2*F*_c_^2^)/3
5404 reflections	(Δ/σ)_max_ = 0.001
367 parameters	Δρ_max_ = 0.78 e Å^−^^3^
60 restraints	Δρ_min_ = −0.44 e Å^−^^3^
0 constraints	

### Special details

**Table:**

Geometry. All esds (except the esd in the dihedral angle between two l.s. planes) are estimated using the full covariance matrix. The cell esds are taken into account individually in the estimation of esds in distances, angles and torsion angles; correlations between esds in cell parameters are only used when they are defined by crystal symmetry. An approximate (isotropic) treatment of cell esds is used for estimating esds involving l.s. planes.

### Fractional atomic coordinates and isotropic or equivalent isotropic displacement parameters (Å^2^)

**Table:**

	*x*	*y*	*z*	*U*_iso_*/*U*_eq_	Occ. (<1)
Ce1	0.44439 (2)	0.34074 (2)	0.44106 (2)	0.02854 (8)	
C11	0.4237 (6)	0.3197 (4)	0.2473 (2)	0.0778 (19)	
C12	0.3779 (5)	0.3321 (3)	0.30394 (19)	0.0538 (13)	
O12	0.4339 (3)	0.3049 (2)	0.34228 (12)	0.0486 (8)	
C13	0.2728 (5)	0.3716 (4)	0.3061 (2)	0.0646 (15)	
H13	0.2427	0.3924	0.2739	0.078*	
C14	0.2092 (4)	0.3819 (4)	0.3546 (2)	0.0600 (14)	
O14	0.2451 (3)	0.3617 (2)	0.40111 (12)	0.0448 (7)	
C15	0.0892 (6)	0.4173 (6)	0.3520 (3)	0.113 (3)	
H15A	0.0766	0.4406	0.3167	0.169*	
H15B	0.0812	0.4584	0.3794	0.169*	
H15C	0.0338	0.3752	0.3583	0.169*	
C21	0.4510 (5)	0.5793 (3)	0.33461 (19)	0.0586 (14)	
C22	0.5053 (4)	0.5033 (3)	0.35843 (18)	0.0439 (10)	
O22	0.4552 (3)	0.47868 (18)	0.40133 (11)	0.0408 (7)	
C23	0.5971 (5)	0.4712 (3)	0.3316 (2)	0.0536 (12)	
H23	0.6201	0.496	0.2994	0.064*	
C24	0.6600 (4)	0.4024 (3)	0.34943 (19)	0.0468 (11)	
O24	0.6358 (2)	0.36304 (19)	0.39146 (12)	0.0429 (7)	
C25	0.7622 (5)	0.3774 (4)	0.3167 (2)	0.0700 (16)	
H25A	0.7489	0.3897	0.279	0.105*	
H25B	0.7745	0.3203	0.3208	0.105*	
H25C	0.8288	0.4063	0.3292	0.105*	
C31	0.1589 (4)	0.4538 (3)	0.5562 (2)	0.0607 (14)	
C32	0.2598 (4)	0.3977 (3)	0.54542 (19)	0.0430 (10)	
O32	0.3247 (3)	0.42071 (18)	0.50708 (12)	0.0454 (7)	
C33	0.2669 (4)	0.3297 (3)	0.5767 (2)	0.0529 (12)	
H33	0.2102	0.3207	0.6027	0.063*	
C34	0.3567 (4)	0.2718 (3)	0.57165 (18)	0.0467 (11)	
O34	0.4301 (3)	0.27381 (19)	0.53500 (12)	0.0448 (7)	
C35	0.3625 (6)	0.2034 (4)	0.6122 (3)	0.0802 (19)	
H35A	0.316	0.2164	0.6433	0.12*	
H35B	0.4407	0.1958	0.6236	0.12*	
H35C	0.3346	0.1546	0.5957	0.12*	
F11B	0.4262 (11)	0.3878 (4)	0.2185 (3)	0.166 (5)	0.829 (14)
F11A	0.3683 (6)	0.2702 (5)	0.2173 (2)	0.132 (3)	0.829 (14)
F11C	0.5308 (6)	0.2914 (7)	0.2481 (2)	0.156 (5)	0.829 (14)
F11D	0.455 (3)	0.2450 (11)	0.2438 (15)	0.131 (14)*	0.171 (14)
F11E	0.503 (2)	0.3703 (15)	0.2358 (11)	0.080 (9)*	0.171 (14)
F11F	0.336 (2)	0.330 (3)	0.2134 (15)	0.149 (17)*	0.171 (14)
F21A	0.3517 (8)	0.5668 (4)	0.3129 (5)	0.148 (4)	0.838 (17)
F21B	0.5143 (11)	0.6165 (5)	0.2974 (4)	0.160 (5)	0.838 (17)
F21C	0.4371 (7)	0.6369 (2)	0.3718 (2)	0.0713 (18)	0.838 (17)
F21D	0.4696 (19)	0.5826 (17)	0.2820 (6)	0.060 (8)*	0.162 (17)
F21E	0.483 (3)	0.6448 (16)	0.3572 (13)	0.108 (15)*	0.162 (17)
F21F	0.3356 (14)	0.5734 (15)	0.3364 (10)	0.054 (7)*	0.162 (17)
F31A	0.1248 (6)	0.4523 (5)	0.6089 (2)	0.137 (3)	0.836 (11)
F31B	0.0676 (4)	0.4319 (3)	0.5295 (3)	0.110 (3)	0.836 (11)
F31C	0.1797 (4)	0.5278 (2)	0.5457 (4)	0.115 (3)	0.836 (11)
F31E	0.129 (3)	0.487 (2)	0.5071 (8)	0.125 (13)*	0.164 (11)
F31F	0.175 (4)	0.5175 (18)	0.5874 (14)	0.16 (2)*	0.164 (11)
F31D	0.065 (2)	0.419 (2)	0.5740 (17)	0.140 (16)*	0.164 (11)
O1W	0.5906 (3)	0.4133 (2)	0.50449 (13)	0.0432 (7)	
H1WA	0.627 (4)	0.455 (2)	0.495 (2)	0.065*	
H1WB	0.574 (5)	0.426 (4)	0.5364 (11)	0.065*	
O2W	0.5781 (3)	0.2200 (2)	0.45895 (15)	0.0536 (9)	
H2WA	0.629 (4)	0.193 (4)	0.442 (2)	0.08*	
H2WB	0.578 (5)	0.201 (4)	0.4904 (13)	0.08*	
O3W	0.3258 (3)	0.2079 (2)	0.44205 (16)	0.0578 (9)	
H3WA	0.270 (4)	0.186 (4)	0.425 (2)	0.087*	
H3WB	0.346 (6)	0.161 (2)	0.451 (3)	0.087*	

### Atomic displacement parameters (Å^2^)

**Table:**

	*U*^11^	*U*^22^	*U*^33^	*U*^12^	*U*^13^	*U*^23^
Ce1	0.02544 (11)	0.03113 (12)	0.02903 (12)	0.00204 (9)	0.00172 (9)	0.00215 (9)
C11	0.104 (6)	0.089 (5)	0.040 (3)	−0.012 (4)	−0.001 (3)	−0.008 (3)
C12	0.066 (3)	0.060 (3)	0.035 (2)	−0.015 (3)	−0.001 (2)	−0.002 (2)
O12	0.0509 (19)	0.059 (2)	0.0355 (16)	0.0012 (16)	0.0016 (14)	−0.0088 (15)
C13	0.068 (4)	0.080 (4)	0.046 (3)	0.005 (3)	−0.012 (3)	0.011 (3)
C14	0.043 (3)	0.076 (4)	0.061 (3)	0.004 (3)	−0.011 (2)	0.013 (3)
O14	0.0316 (15)	0.059 (2)	0.0437 (17)	−0.0006 (14)	−0.0022 (13)	0.0072 (14)
C15	0.066 (4)	0.174 (9)	0.097 (5)	0.054 (5)	−0.018 (4)	0.035 (6)
C21	0.089 (4)	0.046 (3)	0.041 (3)	0.010 (3)	0.004 (3)	0.011 (2)
C22	0.054 (3)	0.038 (2)	0.039 (2)	0.003 (2)	−0.003 (2)	0.0074 (18)
O22	0.0475 (17)	0.0375 (15)	0.0373 (15)	0.0058 (14)	0.0065 (13)	0.0087 (13)
C23	0.061 (3)	0.054 (3)	0.046 (3)	0.006 (2)	0.015 (2)	0.019 (2)
C24	0.036 (2)	0.060 (3)	0.044 (2)	0.004 (2)	0.008 (2)	0.011 (2)
O24	0.0314 (15)	0.0551 (18)	0.0423 (17)	0.0061 (13)	0.0063 (13)	0.0154 (14)
C25	0.055 (3)	0.093 (4)	0.062 (3)	0.014 (3)	0.027 (3)	0.014 (3)
C31	0.040 (3)	0.057 (3)	0.085 (4)	0.000 (2)	0.014 (3)	−0.019 (3)
C32	0.030 (2)	0.049 (3)	0.049 (3)	−0.0027 (19)	0.0042 (19)	−0.016 (2)
O32	0.0467 (17)	0.0472 (17)	0.0424 (16)	0.0084 (15)	0.0108 (14)	−0.0012 (14)
C33	0.046 (3)	0.061 (3)	0.051 (3)	−0.004 (2)	0.021 (2)	−0.001 (2)
C34	0.047 (3)	0.054 (3)	0.039 (2)	−0.008 (2)	0.007 (2)	0.002 (2)
O34	0.0467 (18)	0.0533 (19)	0.0343 (15)	0.0073 (15)	0.0087 (14)	0.0063 (14)
C35	0.095 (5)	0.078 (4)	0.068 (4)	0.007 (4)	0.030 (3)	0.032 (3)
F11B	0.274 (13)	0.152 (7)	0.073 (4)	0.019 (7)	0.064 (6)	0.042 (4)
F11A	0.142 (6)	0.173 (7)	0.082 (4)	−0.010 (5)	−0.013 (4)	−0.076 (4)
F11C	0.099 (5)	0.305 (13)	0.065 (3)	0.041 (7)	0.018 (3)	−0.036 (5)
F21A	0.196 (8)	0.097 (4)	0.151 (7)	0.052 (5)	−0.128 (6)	−0.018 (5)
F21B	0.238 (10)	0.096 (5)	0.146 (7)	0.068 (6)	0.108 (7)	0.087 (5)
F21C	0.105 (5)	0.040 (2)	0.069 (3)	0.012 (2)	−0.006 (3)	0.0032 (19)
F31A	0.129 (6)	0.175 (7)	0.106 (5)	0.071 (5)	0.053 (4)	−0.015 (4)
F31B	0.049 (3)	0.090 (4)	0.191 (7)	0.017 (2)	−0.034 (3)	−0.047 (4)
F31C	0.075 (3)	0.042 (2)	0.228 (9)	0.005 (2)	0.055 (4)	−0.021 (3)
O1W	0.0436 (17)	0.0465 (18)	0.0396 (17)	−0.0101 (14)	−0.0018 (14)	−0.0037 (15)
O2W	0.048 (2)	0.059 (2)	0.054 (2)	0.0261 (16)	0.0161 (16)	0.0169 (17)
O3W	0.059 (2)	0.0420 (18)	0.072 (2)	−0.0178 (17)	−0.0242 (19)	0.0094 (17)

### Geometric parameters (Å, º)

**Table:**

Ce1—O22	2.481 (3)	C22—O22	1.271 (5)
Ce1—O12	2.500 (3)	C22—C23	1.362 (7)
Ce1—O32	2.512 (3)	C23—C24	1.420 (7)
Ce1—O14	2.542 (3)	C23—H23	0.93
Ce1—O34	2.563 (3)	C24—O24	1.252 (5)
Ce1—O24	2.565 (3)	C24—C25	1.493 (6)
Ce1—O2W	2.566 (3)	C25—H25A	0.96
Ce1—O3W	2.592 (3)	C25—H25B	0.96
Ce1—O1W	2.599 (3)	C25—H25C	0.96
C11—F11A	1.276 (7)	C31—F31C	1.273 (7)
C11—F11E	1.280 (15)	C31—F31B	1.299 (6)
C11—F11D	1.289 (16)	C31—F31D	1.308 (17)
C11—F11B	1.328 (8)	C31—F31F	1.315 (17)
C11—F11F	1.330 (16)	C31—F31A	1.354 (7)
C11—F11C	1.331 (8)	C31—F31E	1.372 (16)
C11—C12	1.504 (8)	C31—C32	1.518 (6)
C12—O12	1.230 (6)	C32—O32	1.266 (5)
C12—C13	1.387 (8)	C32—C33	1.365 (7)
C13—C14	1.413 (8)	C33—C34	1.422 (7)
C13—H13	0.93	C33—H33	0.93
C14—O14	1.262 (6)	C34—O34	1.241 (5)
C14—C15	1.514 (8)	C34—C35	1.507 (7)
C15—H15A	0.96	C35—H35A	0.96
C15—H15B	0.96	C35—H35B	0.96
C15—H15C	0.96	C35—H35C	0.96
C21—F21E	1.273 (15)	O1W—H1WA	0.84 (2)
C21—F21A	1.289 (8)	O1W—H1WB	0.83 (2)
C21—F21D	1.312 (14)	O2W—H2WA	0.85 (2)
C21—F21B	1.324 (7)	O2W—H2WB	0.84 (2)
C21—F21C	1.329 (6)	O3W—H3WA	0.85 (2)
C21—F21F	1.347 (15)	O3W—H3WB	0.84 (2)
C21—C22	1.522 (6)		
			
O22—Ce1—O12	80.70 (11)	F21A—C21—F21B	106.6 (7)
O22—Ce1—O32	78.42 (10)	F21A—C21—F21C	106.8 (6)
O12—Ce1—O32	136.35 (11)	F21B—C21—F21C	102.1 (6)
O22—Ce1—O14	76.68 (10)	F21E—C21—F21F	109.9 (15)
O12—Ce1—O14	67.23 (10)	F21D—C21—F21F	101.5 (12)
O32—Ce1—O14	70.85 (10)	F21E—C21—C22	114.3 (17)
O22—Ce1—O34	138.85 (10)	F21A—C21—C22	113.6 (5)
O12—Ce1—O34	140.17 (11)	F21D—C21—C22	110.2 (13)
O32—Ce1—O34	67.04 (10)	F21B—C21—C22	114.7 (5)
O14—Ce1—O34	110.31 (10)	F21C—C21—C22	112.1 (4)
O22—Ce1—O24	68.74 (10)	F21F—C21—C22	110.0 (12)
O12—Ce1—O24	67.40 (10)	O22—C22—C23	129.5 (4)
O32—Ce1—O24	135.42 (10)	O22—C22—C21	113.1 (4)
O14—Ce1—O24	126.11 (10)	C23—C22—C21	117.4 (4)
O34—Ce1—O24	123.05 (9)	C22—O22—Ce1	130.0 (3)
O22—Ce1—O2W	138.56 (11)	C22—C23—C24	124.5 (4)
O12—Ce1—O2W	90.68 (12)	C22—C23—H23	117.7
O32—Ce1—O2W	129.33 (11)	C24—C23—H23	117.7
O14—Ce1—O2W	136.45 (12)	O24—C24—C23	123.6 (4)
O34—Ce1—O2W	63.28 (10)	O24—C24—C25	118.7 (4)
O24—Ce1—O2W	70.52 (10)	C23—C24—C25	117.7 (4)
O22—Ce1—O3W	143.87 (11)	C24—O24—Ce1	131.4 (3)
O12—Ce1—O3W	77.44 (12)	C24—C25—H25A	109.5
O32—Ce1—O3W	98.26 (12)	C24—C25—H25B	109.5
O14—Ce1—O3W	68.46 (11)	H25A—C25—H25B	109.5
O34—Ce1—O3W	65.93 (11)	C24—C25—H25C	109.5
O24—Ce1—O3W	126.04 (12)	H25A—C25—H25C	109.5
O2W—Ce1—O3W	70.35 (13)	H25B—C25—H25C	109.5
O22—Ce1—O1W	77.26 (10)	F31C—C31—F31B	108.7 (6)
O12—Ce1—O1W	136.27 (11)	F31D—C31—F31F	105.9 (17)
O32—Ce1—O1W	74.57 (11)	F31C—C31—F31A	105.5 (5)
O14—Ce1—O1W	139.99 (11)	F31B—C31—F31A	103.7 (6)
O34—Ce1—O1W	72.65 (10)	F31D—C31—F31E	105.0 (16)
O24—Ce1—O1W	69.51 (10)	F31F—C31—F31E	103.0 (16)
O2W—Ce1—O1W	81.87 (12)	F31C—C31—C32	113.8 (4)
O3W—Ce1—O1W	137.14 (11)	F31B—C31—C32	112.0 (4)
F11E—C11—F11D	113.8 (16)	F31D—C31—C32	115.9 (17)
F11A—C11—F11B	104.2 (7)	F31F—C31—C32	118.7 (19)
F11E—C11—F11F	109.5 (15)	F31A—C31—C32	112.5 (5)
F11D—C11—F11F	107.7 (16)	F31E—C31—C32	106.8 (14)
F11A—C11—F11C	104.9 (7)	O32—C32—C33	129.0 (4)
F11B—C11—F11C	106.6 (8)	O32—C32—C31	114.2 (4)
F11A—C11—C12	116.3 (6)	C33—C32—C31	116.8 (4)
F11E—C11—C12	111.8 (13)	C32—O32—Ce1	130.8 (3)
F11D—C11—C12	106.9 (17)	C32—C33—C34	123.3 (4)
F11B—C11—C12	112.7 (6)	C32—C33—H33	118.4
F11F—C11—C12	106.7 (19)	C34—C33—H33	118.4
F11C—C11—C12	111.4 (5)	O34—C34—C33	123.5 (4)
O12—C12—C13	127.6 (5)	O34—C34—C35	117.9 (5)
O12—C12—C11	118.1 (5)	C33—C34—C35	118.6 (4)
C13—C12—C11	114.3 (5)	C34—O34—Ce1	135.1 (3)
C12—O12—Ce1	133.0 (3)	C34—C35—H35A	109.5
C12—C13—C14	123.4 (5)	C34—C35—H35B	109.5
C12—C13—H13	118.3	H35A—C35—H35B	109.5
C14—C13—H13	118.3	C34—C35—H35C	109.5
O14—C14—C13	123.9 (5)	H35A—C35—H35C	109.5
O14—C14—C15	116.4 (5)	H35B—C35—H35C	109.5
C13—C14—C15	119.6 (5)	Ce1—O1W—H1WA	123 (4)
C14—O14—Ce1	133.5 (3)	Ce1—O1W—H1WB	122 (4)
C14—C15—H15A	109.5	H1WA—O1W—H1WB	100 (5)
C14—C15—H15B	109.5	Ce1—O2W—H2WA	138 (4)
H15A—C15—H15B	109.5	Ce1—O2W—H2WB	117 (4)
C14—C15—H15C	109.5	H2WA—O2W—H2WB	105 (6)
H15A—C15—H15C	109.5	Ce1—O3W—H3WA	140 (5)
H15B—C15—H15C	109.5	Ce1—O3W—H3WB	129 (5)
F21E—C21—F21D	110.2 (15)	H3WA—O3W—H3WB	87 (6)
			
F11A_a—C11—C12—O12	109.7 (8)	F21C—C21—C22—C23	−130.5 (6)
F11E—C11—C12—O12	−79.3 (17)	F21F—C21—C22—C23	137.2 (12)
F11D—C11—C12—O12	45.9 (19)	C23—C22—O22—Ce1	−24.8 (8)
F11B—C11—C12—O12	−130.0 (8)	C21—C22—O22—Ce1	154.3 (3)
F11F—C11—C12—O12	161.0 (19)	O22—C22—C23—C24	−3.3 (9)
F11C—C11—C12—O12	−10.3 (9)	C21—C22—C23—C24	177.6 (5)
F11A—C11—C12—C13	−68.2 (9)	C22—C23—C24—O24	1.5 (9)
F11E—C11—C12—C13	102.7 (16)	C22—C23—C24—C25	−177.0 (5)
F11D—C11—C12—C13	−132.0 (19)	C23—C24—O24—Ce1	27.8 (7)
F11B—C11—C12—C13	52.0 (10)	C25—C24—O24—Ce1	−153.7 (4)
F11F—C11—C12—C13	−17.0 (19)	F31C—C31—C32—O32	−30.8 (8)
F11C—C11—C12—C13	171.7 (8)	F31B—C31—C32—O32	92.9 (7)
C13—C12—O12—Ce1	−25.9 (8)	F31D—C31—C32—O32	148 (2)
C11—C12—O12—Ce1	156.4 (4)	F31F—C31—C32—O32	−84 (2)
O12—C12—C13—C14	−4.5 (10)	F31A—C31—C32—O32	−150.7 (6)
C11—C12—C13—C14	173.2 (6)	F31E—C31—C32—O32	31.9 (18)
C12—C13—C14—O14	5.5 (10)	F31C—C31—C32—C33	150.7 (6)
C12—C13—C14—C15	−173.0 (7)	F31B—C31—C32—C33	−85.6 (7)
C13—C14—O14—Ce1	23.3 (9)	F31D—C31—C32—C33	−30 (2)
C15—C14—O14—Ce1	−158.2 (5)	F31F—C31—C32—C33	98 (2)
F21E—C21—C22—O22	82.1 (19)	F31A—C31—C32—C33	30.8 (7)
F21A—C21—C22—O22	−70.9 (8)	F31E—C31—C32—C33	−146.6 (17)
F21D—C21—C22—O22	−153.2 (11)	C33—C32—O32—Ce1	28.9 (7)
F21B—C21—C22—O22	166.1 (9)	C31—C32—O32—Ce1	−149.4 (3)
F21C—C21—C22—O22	50.2 (7)	O32—C32—C33—C34	2.2 (8)
F21F—C21—C22—O22	−42.1 (12)	C31—C32—C33—C34	−179.6 (5)
F21E—C21—C22—C23	−98.6 (19)	C32—C33—C34—O34	−7.2 (8)
F21A—C21—C22—C23	108.4 (9)	C32—C33—C34—C35	173.8 (5)
F21D—C21—C22—C23	26.1 (13)	C33—C34—O34—Ce1	−19.7 (7)
F21B—C21—C22—C23	−14.6 (11)	C35—C34—O34—Ce1	159.3 (4)

### Hydrogen-bond geometry (Å, º)

**Table:**

*D*—H···*A*	*D*—H	H···*A*	*D*···*A*	*D*—H···*A*
O1*W*—H1*WA*···O32^i^	0.84 (2)	2.13 (3)	2.927 (4)	158 (5)
O1*W*—H1*WB*···O22^i^	0.83 (2)	2.23 (4)	2.969 (4)	149 (6)
O2*W*—H2*WA*···O14^ii^	0.85 (2)	1.91 (2)	2.759 (4)	177 (6)
O3*W*—H3*WA*···O24^iii^	0.85 (2)	1.94 (2)	2.792 (4)	176 (7)

Symmetry codes: (i) −*x*+1, −*y*+1, −*z*+1; (ii) *x*+1/2, −*y*+1/2, *z*; (iii) *x*−1/2, −*y*+1/2, *z*.
